# CRISPR/Cas9-engineering of HMC-1.2 cells renders a human mast cell line with a single D816V-KIT mutation: An improved preclinical model for research on mastocytosis

**DOI:** 10.3389/fimmu.2023.1078958

**Published:** 2023-03-21

**Authors:** Geethani Bandara, Guido H. Falduto, Andrea Luker, Yun Bai, Annika Pfeiffer, Justin Lack, Dean D. Metcalfe, Ana Olivera

**Affiliations:** ^1^ Mast Cell Biology Section, Laboratory of Allergic Diseases, National Institute of Allergy and Infectious Diseases, National Institutes of Health, Bethesda, MD, United States; ^2^ National Institute of Allergy and Infectious Diseases (NIAID), Collaborative Bioinformatics Resource (NCBR), National Institute of Allergy and Infectious Diseases, National Institutes of Health, Bethesda, MD, United States

**Keywords:** KIT variants, D816V-KIT, neoplastic human mast cells, mastocytosis, mast cell survival, HMC-1.2 cells

## Abstract

The HMC-1.2 human mast cell (huMC) line is often employed in the study of attributes of neoplastic huMCs as found in patients with mastocytosis and their sensitivity to interventional drugs *in vitro* and *in vivo*. HMC-1.2 cells express constitutively active KIT, an essential growth factor receptor for huMC survival and function, due to the presence of two oncogenic mutations (D816V and V560G). However, systemic mastocytosis is commonly associated with a single D816V-KIT mutation. The functional consequences of the coexisting KIT mutations in HMC-1.2 cells are unknown. We used CRISPR/Cas9-engineering to reverse the V560G mutation in HMC-1.2 cells, resulting in a subline (HMC-1.3) with a single mono-allelic D816V-KIT variant. Transcriptome analyses predicted reduced activity in pathways involved in survival, cell-to-cell adhesion, and neoplasia in HMC-1.3 compared to HMC-1.2 cells, with differences in expression of molecular components and cell surface markers. Consistently, subcutaneous inoculation of HMC-1.3 into mice produced significantly smaller tumors than HMC-1.2 cells, and in colony assays, HMC-1.3 formed less numerous and smaller colonies than HMC-1.2 cells. However, in liquid culture conditions, the growth of HMC-1.2 and HMC-1.3 cells was comparable. Phosphorylation levels of ERK1/2, AKT and STAT5, representing pathways associated with constitutive oncogenic KIT signaling, were also similar between HMC-1.2 and HMC-1.3 cells. Despite these similarities in liquid culture, survival of HMC-1.3 cells was diminished in response to various pharmacological inhibitors, including tyrosine kinase inhibitors used clinically for treatment of advanced systemic mastocytosis, and JAK2 and BCL2 inhibitors, making HMC-1.3 more susceptible to these drugs than HMC-1.2 cells. Our study thus reveals that the additional V560G-KIT oncogenic variant in HMC-1.2 cells modifies transcriptional programs induced by D816V-KIT, confers a survival advantage, alters sensitivity to interventional drugs, and increases the tumorigenicity, suggesting that engineered huMCs with a single D816V-KIT variant may represent an improved preclinical model for mastocytosis.

## Introduction

KIT (CD117) is a transmembrane tyrosine kinase receptor critical for mast cell survival, proliferation, homing and responsiveness (1–3). Activation of KIT normally occurs by binding to its ligand stem cell factor (SCF), which induces KIT dimerization and activation of its kinase domain that then trans-phosphorylates multiple tyrosine residues in the receptor resulting in recruitment and activation of signaling cascades (4). Various gain-of-function polymorphisms in KIT can also cause KIT dimerization and tyrosine kinase activation in the absence of ligand (4–6). These activating KIT variants, mostly somatic, have been associated with human malignancies, including mast cell proliferative disorders such as mastocytosis (1, 4). In patients with mastocytosis, mast cells accumulate in the tissues, and increased levels of mast cell mediators are thought to contribute to the symptoms associated with this disease (such as recurrent episodes of flushing, shortness of breath, abdominal pain, etc.) (7).

**Figure 1:**
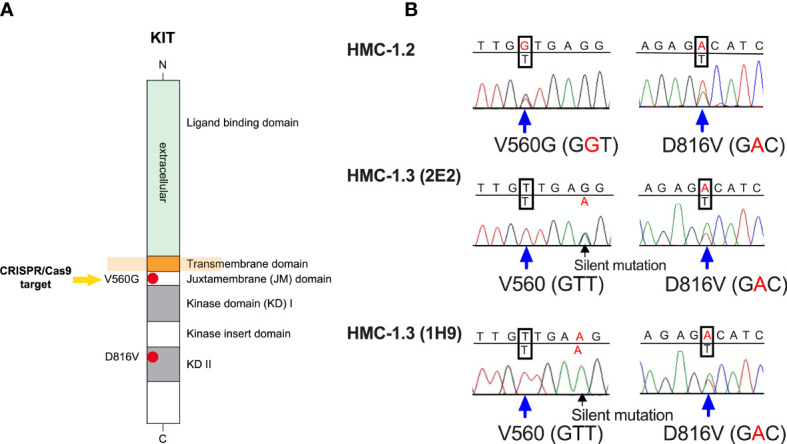
The huMC line HMC-1.3 generated by CRISPR/Cas9-mediated editing of HMC-1.2 cells has a single D816V-KIT mutation. **(A)** Schematic representation of the structure of KIT, highlighting the presence in HMC-1.2 cells of the oncogenic V560G variant in the juxtamembrane (JM) domain and of D816V-KIT in the kinase domain (KD). The arrow indicates the target mutation for CRISPR/Cas9 editing. **(B)** Representative Sanger sequencing chromatograms of KIT exons from HMC-1.2 or two separate clones (1H9 and 2E2) of HMC-1.3 cells, demonstrating heterozygous presence of D816V in both HMC-1.2 and in HMC-1.3, and a heterozygous V560G mutation in HMC-1.2 (GGT) that was reversed to the normal allele on this site (GTT) after the CRISPR/Cas9-mediated editing in HMC-1.3.

Most oncogenic KIT variants identified in human neoplasms occur in mutational hot spots. In more than 80% of adult patients with systemic mastocytosis, KIT mutations are found in the kinase domain (KD) of KIT (exon 17), commonly in position 816 (D816V), while in childhood mastocytosis D816V has a lower incidence, and a number of mutations occur in the extracellular domain of KIT (exons 8 and 9). Mutations in the juxtamembrane (JM) domain of KIT such as V560G (exon 11) are rare in mastocytosis but frequent in gastrointestinal tumors (GIST) (1, 4, 8) (see **Figure 1A**). Variants in the KD may differ quantitatively and qualitatively from those in the JM domain, particularly regarding the oncogenic signaling pathways they induce, transforming potential (4) and sensitivity to inhibitors of KIT or signaling pathways, a feature of clinical significance for diagnosis and treatment of disease (8, 9).

In recent years, novel tyrosine kinase inhibitors as well as other strategies have been tested to reduce KIT activity in hematological neoplasms, with some candidates approved and others in clinical trials. However, there is still an unmet medical need for approaches that may reduce mast cell burden/activity in these disorders (9–11). One widely used preclinical model for the evaluation of potential treatments is the human mast cell line HMC-1, isolated from a patient with mast cell leukemia (12, 13). Subsequently, two sublines within HMC-1 cultures were identified: HMC-1.1 cells, containing a heterozygous mutation in V560G-KIT; and HMC-1.2 cells, containing the V560G and D816V-KIT mutations (14) (**Figure 1A**). The use of HMC-1 mast cells as a pre-clinical model (15) has several advantages: (-i-) the expression of KIT variants frequently found in mastocytosis and other diseases; (-ii-) rapid growth in culture, which allows for high-throughput pharmacological studies; (-iii-) development of tumors in xenograft mouse models for *in vivo* drug testing; and (-iv-) commercial availability. However, a drawback of this model is that neither HMC-1.1 or HMC-1.2 cells carry only the D816V mutation. HMC-1.2 cells which do carry D816V-KIT also carry V560G-KIT and, thus, it is unknown to what extent the signaling pathways, cell responses or sensitivity to drugs are affected by the combined effects of the two KIT variants. Although the presence of more than one KIT mutation has been described in patients with childhood mastocytosis (16) and in instances of acute myeloid leukemia (17) and GIST refractory to treatment (18), most patients with systemic mastocytosis present with D816V-KIT alone (1, 16), emphasizing the need for a neoplastic mast cell model expressing KIT with the single D816V-KIT mutation.

With these considerations, we employed clustered regularly interspaced short palindromic repeats (CRISPR)/CRISPR-associated protein 9 (Cas9) engineering to correct the V560G mutation in HMC-1.2 cells and compared cell attributes between the resulting cell line and the parental line to investigate potential combinatorial effects of the KIT variants. The CRISPR/Cas9 approach successfully delivered a new subline (HMC-1.3) with the single mono-allelic D816V-KIT of the parental cell line but lacking V560G-KIT. As will be shown, we found that the removal of V560G in D816V-containing huMCs did not significantly affect cell growth or constitutive signaling under normal culture conditions compared to the parental HMC-1.2 but resulted in altered transcriptional programs and reduced tumor growth and metastasis in a xenograft model. The gene expression changes resulted in a higher propensity to cell death in HMC-1.3 cells that was modest under homeostatic conditions but enhanced in the presence of certain pharmacological inhibitors of KIT and signaling targets, such as inhibitors of JAK2, making them more sensitive to these drugs. These results suggest that the presence of V560G-KIT acts as a functional modifier in D816V-KIT mutated cells, enhancing transcriptional programs of survival and potentially other molecular functions. This data is consistent with the conclusion that the engineered HMC-1.3 cell line with a single D816V-KIT variant represents an additional and improved preclinical model for mastocytosis.

## Materials and methods

### Reagents

Antibodies against phospho-AKT(Ser473), ERK, phospho-ERK(Thr202/Tyr204), STAT5, and phospho-STAT5 were obtained from Cell Signaling Technology (Danvers, MA); anti-KIT from R&D Systems (Minneapolis, MN) and Santa Cruz Biotechnology (Santa Cruz, CA); anti-phospho-KIT(Tyr823) from Invitrogen (Carlsbad, CA); anti-AKT from BD Biosciences (San Diego, CA); and anti-β actin from Sigma Aldrich (St. Louis, MO); and anti-rabbit IgG 800CW and anti-mouse IgG 680 RD from Licor Biosciences (Lincoln, NE). Human TruStain FcX and APC-conjugated anti-human c-KIT (clone 104D2), unlabeled anti-human KIT antibody (clone 104D2) and Zombie Aqua fixable viability dye were obtained from Biolegend (San Diego, CA); KIT antibody for IHC from Dako-Agilent (Santa Clara, CA); human SCF from R & D Systems; avapritinib, imatinib, dasatinib, midostaurin, fedratinib, STAT5-IN-1, SH4-54, venetoclax, ripretinib (DCC-2618), ruxolitinib, tofacitinib, and C188-9 from SelleckChem (Houston, TX); LY294002 and U0126 from Tocris (Minneapolis, MN); Pierce BCA assay and CyQuant cell proliferation assay from Thermo Fisher Scientific (Waltham, MA); and ViaStain propidium iodide (PI) staining solution from Nexelom Biosciences (Lawrence, MA).

### Cell cultures

HMC-1.1 and HMC-1.2 were kindly provided by Dr. Butterfield at the Mayo Clinic (12) under a Uniform Biological Material Transfer Agreement. HMC-1.3 were modified from the parental HMC-1.2 as explained below. All sublines were cultured in IMDM medium supplemented with FBS (10%), l-glutamine (2 mM), penicillin (100 units/ml), and streptomycin (100 µg/ml) and were refreshed from an earlier stock every 3 months.

### Genome editing of the KIT-V560G variant in HMC-1.2 using CRISPR/Cas9

Genome editing of HMC-1.2 to revert the heterozygous single nucleotide G560 mutation in KIT (G, GGT) to WT (V, GTT) (**Figure 1A**) was done by Applied StemCell (Milpitas, CA). A proprietary CRISPR/Cas9 genome editing technology was employed following the steps illustrated in **Supplementary Figure 1**. Two guide RNAs (gRNAs) were designed using a proprietary gRNA design tool by Applied Stem Cell. Based on the proximity to the target site, off-target profile and predicted activity, the gRNA “Kitex11g2” (5′- AGTACAGTGGAAGGTTGGTG -3′) was selected. The single-stranded oligodeoxynucleotide donor (ssODN) was designed to be used as a repair template for generating the desired GGT>GTT point mutation flanked by 5’ and 3’ homology arms for the targeted region to the gRNA cut sites during the homology-directed repair process (ssODN sequence: 5’-TGTTCTCTCTCCAGAGTGCTCTAATGACTGAGACAATAATTATTAAAAGGTGATCTATTTTTCCCTTTCTCCCCACAGAAACCCATGTATGAAGTACAGTGGAAGGTTGTTGAAGAGATAAATGGAAACAATTATGTTTACATAGACCCAACACAACTTCCTTATGATCACAAATGGGAGTTTCCCAGAAACAGGCTGA- 3’). In the design of the ssODN, a silent mutation (GAG to GAA, corresponding to aminoacidic position 561) was introduced to destroy a PAM site and prevent re-cutting in the modified genome after repair. Double-stranded gRNA was inserted between the two Bbs1 sites into a bicistronic vector (pBT-U6-Cas9–2A-GFP) for co-expression with the Cas9 protein (pBT-U6-Cas9–2A-GFP). The resulting plasmid was transfected by electroporation together with the ss-ODN into HMC-1.2 cells using 1500 V, 10 ms, and 3 ps in the Neon Transfection System (Thermo Fisher Scientific). Cells were cultured for 48 h, and then selected by puromycin (1 μg/ml) for two to three weeks. The resulting cell population was then further subjected to limiting dilution for cloning and genotype analysis. Fifty single clones were genotyped around the 560 and 816 regions, and 16 were confirmed with the predicted WT sequence in position 560 but containing the mono-allelic D816V variant like the parental line. Sanger sequencing of all KIT exons in five of the clones further confirmed that no other areas in KIT, except those intended, differed from the parental KIT sequence.

### Western blots

Cell lysates for Western blots were prepared from 1x10^6^ cells in RIPA buffer as described (19). Protein content in each sample was determined by Pierce BCA assay for equal loading. To assess the effect of SCF on signaling, cells were serum-starved for 2 h. After rinsing twice and resuspending in HEPES buffer containing 0.04% BSA (20), cells were activated with human SCF (100 ng/mL) for the indicated times. Cell protein extracts (20 µg) were resolved by SDS-PAGE (4-12% acrylamide gradient gels) and transferred to nitrocellulose membranes. Membranes were blocked for 60 min with Odyssey blocking buffer (LI-COR Biosciences) and incubated overnight with the primary antibodies (1:1000 dilution). Blots were washed and after 60 min of incubation with the IR-dye (680RD or 800CW)-labeled secondary antibodies, immunoreactive proteins were visualized using an Odyssey CLx imager (LI-COR Biosciences).

### Determination of cell surface and intracellular KIT by FACS

Cells (1x10^6^) were serum-starved for 2 h and then immediately resuspended in cold Azide-containing FACS buffer and placed on ice for 15 min to minimize receptor turn-over. All cells were stained with Zombie Aqua Fixable Viability dye and Fc receptors blocked with Human TruStain FcX according to manufacturer’s instructions. Cells were then divided among three tubes for surface, intracellular, and total KIT staining. To quantify intracellular KIT, the cell surface was first saturated with unlabeled anti-human KIT antibody, followed by fixation, permeabilization, and intracellular staining with the same clone of APC-conjugated anti-human KIT. To quantify surface KIT, intact cells were stained with APC-conjugated KIT antibody; these samples were similarly fixed and permeabilized to maintain consistent scatter profiles. To quantify total KIT, intact cells were first stained with APC-conjugated KIT and then fixed/permeabilized for intracellular staining with APC-conjugated KIT. This measured total mean fluorescence intensity (MFI) was statistically indistinguishable from the summative value of Surface MFI + Intracellular MFI using a student t-test, thus validating the staining protocol. Each staining step was performed by incubating the cells on ice for 20 min with 0.5 µg of antibody. Data acquisition was performed with a LSRFortessa™ Cell Analyzer (BD Biosciences) and analyzed using FlowJo (v10) software. The proportion of KIT on the cell surface was calculated as follows:


$$\%\text{Surface KIT}=\frac{Surface\;MFI}{\left(Surface\;MFI\right)+\left(Intracellular\;MFI\right)}\times 100$$

### RNA isolation, RNA seq and transcriptome analysis

HMC-1.1, HMC-1.2 and the two individual clones of HMC-1.3 cells (1H9 and 2E2) were plated (3x10^5^) in triplicates in 6 well plates. After 72 h, total RNA from 2 × 10^6^ cells was extracted using RNeasy plus mini kit (Qiagen, Valencia, CA) and treated with DNase (Bio-Rad, Hercules, CA) to eliminate gDNA contamination. 600 ng of total RNA from each of the 12 samples (4 cell lines done in triplicates) was used as input for an mRNA capture with oligo-dT coated magnetic beads. The mRNA was fragmented, and then a random-primed cDNA synthesis was performed. The resulting double-strand cDNA were pooled and sequenced on NextSeq2000 using Illumina Stranded mRNA Prep Ligation Library Prep and paired-end sequencing. The samples had 67 to 99 million pass filter reads with more than 94% of bases above the quality score of Q30. Samples were processed from raw fastq through to raw expression values using the RNA-seek workflow (https://github.com/OpenOmics/RNA-seek). Within that workflow, raw reads were trimmed for adapters and low-quality bases using Cutadapt v2.10 (21) before alignment to the human reference genome (hg38) and the Gencode Release 39 annotation using STAR v2.5.3 in two-pass mode (22). PCR duplicates were marked using Picard MarkDuplicates v2.27.3 (https://broadinstitute.github.io/picard/). The average mapping rate of all samples was 97%, and unique alignment was above 89%. The mapping statistics were calculated using Picard software. Percent coding bases were between 57-59%. Percent UTR bases were 35-36%, and mRNA bases were between 93-94% for all the samples. The samples had 69-72% non-duplicate reads. From the alignment files, gene-level quantification was performed using RSEM v1.3.0 (23) and pairwise differential expression was performed using DESeq2 (24) implemented in iDEP v94 (25). Raw and normalized expression matrices were uploaded to the Gene Expression Omnibus (GEO) and are available under the accession ID# GSE216446 (https://www.ncbi.nlm.nih.gov/geo/query/acc.cgi?acc=GSE216446). Data were also analyzed with the use of QIAGEN IPA (QIAGEN Inc., https://digitalinsights.qiagen.com/IPA) (26).

### qPCR and droplet digital PCR

Total RNA was extracted from 1-3 × 10^6^ cells using RNeasy plus mini kit (Qiagen). To quantify various gene transcripts in the samples, one-step reverse transcription and real time-qPCR reactions were performed using iTaq Universal Probes One-Step Kit and the PrimePCR™ probe sets: MCL1 (qHsaCEP0039327) and BCL2L11 (BIM) (qHsaCEP0025251) (Bio Rad). GAPDH (qHsaCEP0041396) and ACTB (qHsaCEP0036280) were used as reference genes.

For ddPCR determinations of allelic frequency of D816V-KIT, genomic DNA was extracted from 1×10^6^ cells using a QIAamp DNA blood mini kit (Qiagen). PCR amplifications were performed in nanoliter sized droplets generated using a manual droplet generator (Bio-Rad) after mixing the samples with a primer/probe set (PrimePCR ddPCR mutation assay kit, Bio-Rad) following the manufacturer’s instructions. This set is designed to distinguish wild-type KIT from D816V-KIT. Droplets were analyzed on a QX200 Droplet Reader (Bio-Rad).

### Determination of viable and dead cells in liquid cultures

HMC-1 cells (2.5×10^4^ in in 100 µL) were plated in black clear-bottom 96 well plates (Fisher Scientific Pittsburgh, PA) in normal media in the presence or absence of the indicted inhibitors. After 72 h, or as indicated in the figures, 10 µL of ViaStain propidium iodine (PI) staining solution (Nexelom Biosciences, Lawrence, MA) was added to detect dead cells. Total and dead cells were counted using a Celigo Image Cytometer automated system (Nexelom Biosciences). The number of viable cells was calculated by subtracting the number of dead cells from the total numbers. In some experiments, cells were plated in serum-free media and the number of viable and dead cells determined over time as above.

In other experiments, the number of viable cells was determined using a fluorometric CyQuant assay. Cells were plated as above and after 48 or 72 h, an equal volume of 2X CyQuant^®^Direct detection reagent (Thermo Fisher Scientific) containing a fluorescent nucleic acid stain for viable cells was added to the wells to determine cell growth. After 1 h at 37°C, fluorescence was measured at 480/535 nm.

### Cell growth in semi-solid media

For colony assays, 7.5x10^4^ cells were mixed with complete media containing 0.5% Bacto agar (soft agar) and plated in 48 well plates. Alternatively, cells were similarly embedded into Matrigel (Corning Incorporated, Tewksbury, MA) diluted 1:1 in cell culture media following the manufacturer’s protocol. After 6 days, colonies were counted using a Celigo Imager Cytometer.

### Xenograft model of aggressive systemic mastocytosis

NSG mice (NOD.Cg-Prkdcscid Il2rgtm1Wjl/SzJ, J; stock number 005557) were obtained from The Jackson Laboratory and housed for the length of the experiments in a vivarium of the National Institute of Allergy and Infectious Diseases (NIAID), accredited by the American Association for the Accreditation of Laboratory Animal Care (AAALAC). The protocols with live mice were performed under an animal study proposal (LAD2E) approved by the NIAID Division of Intramural Research (DIR) Animal Care and Use Committee under the guidance of the Office of Animal Care and Use of the National Institutes of Health.

Eight-week-old male NSG mice were injected subcutaneously with 1 × 10^6^ HMC-1.2 or HMC-1.3 cells into the right flank. The tumors were allowed to establish until they reached a volume of 50 mm^3^ (usually 18-25 days after injection). From this point, considered Day 0, caliper (Mitutoyo, Japan) measurements were taken daily and the solid tumor volume was calculated as volume (mm^3^) = (length × width^2^)/2 (27). On Day 14, the mice were euthanized for tissue collection. Tumor, spleen, and liver were weighed and fixed in 10% neutral buffered formalin overnight, transferred to 70% ethanol and paraffin embedded. Slides were prepared for histopathology by Histoserv Inc. (Germantown, MD). Slides were deparaffinized and hydrated before antigen retrieval at pH 9. After blocking with hydrogen peroxide, slides were incubated with 1:200 dilution of anti-KIT (CD117) antibody at room temperature followed by incubation with an HRP-conjugated goat-anti-rabbit secondary antibody. Slides were developed using 3,3’-Diaminobenzidine and counterstained with eosin.

### Statistical analysis

Statistical analyses were performed using GraphPad Prism software (version 9.3.1). Unpaired Student’s t tests and 2-way ANOVA tests were used to determine statistical significance as specified in the legends to figures. A *p* value of less than 0.05 was considered significant. Each experiment was done in triplicates or quadruplicates and experiments were repeated at least 3 independent times. Data are shown as mean ± SD or SEM of n≥3 independent experiments as indicated in the figure legends.

## Results

### Correction of the V560G mutation in HMC-1.2 cells by CRISPR/Cas9 engineering generated a huMC line with a single D816V-KIT mutation

After gene editing with CRISPR/Cas9 (**Supplementary Figure 1**), we isolated 16 individual clones with a correct reversal of the mono-allelic mutation in position 560 from GGT (mutated allele) to GTT (normal allele) in the JM domain of KIT (**Figure 1A**). Five of these clones were randomly selected to confirm by Sanger’s sequencing that the complete exon sequences of KIT matched that of the parental HMC-1.2 cells except for the intended base pair conversions (**Figure 1B**). The resulting mast cell clones, containing a single D816V-KIT mutation instead of the double D816V- plus V560G-KIT mutations in the parental cell line, were named HMC-1.3. Two clones (2E2 and 1H9) were chosen for further studies, as they exhibited growth characteristics representative of the average growth rate of all five individual clones (**Supplementary Figure 2**).

The allelic frequency of D816V tested by ddPCR in the sublines was 0% for HMC-1.1 cells and ~50% for HMC-1.2 (50.0 ± 3.3; mean ± SD, n=6), HMC-1.3 (2E2) (51.1 ± 2.8; mean ± SD, n=4) and HMC-1.3 (1H9) (50.5 ± 0.4; mean ± SD, n=4), consistent with the expected mono-allelic presence of D816V in all the corresponding sublines.

### HMC-1.3 cells show similar growth characteristics, cellular distribution of KIT and signaling as the parental HMC-1.2 cells

**Figure 2:**
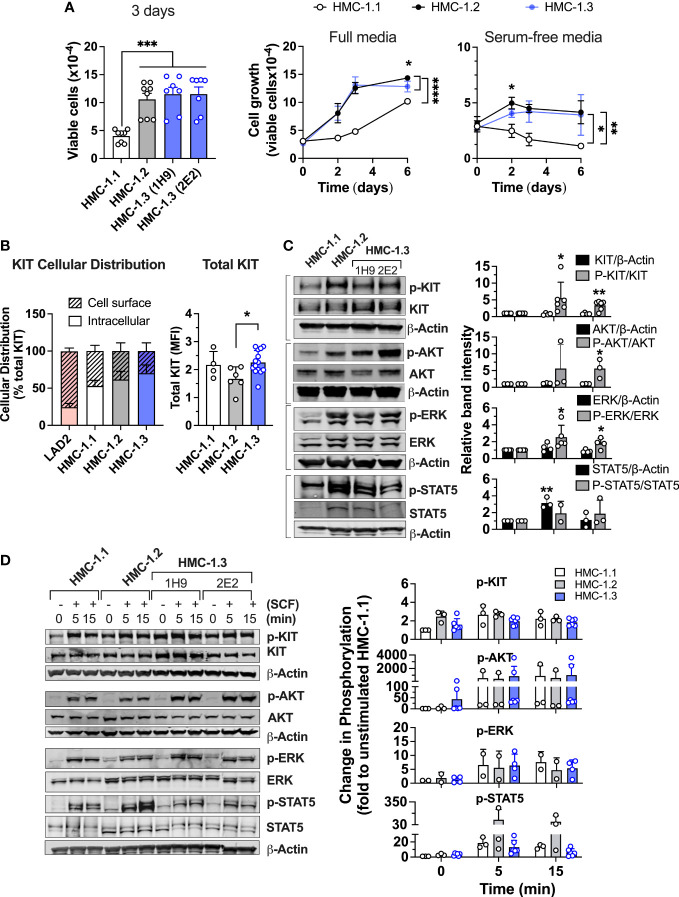
Comparison of cell growth, KIT expression and oncogenic KIT signaling in the various HMC-1 sublines. **(A)** Cell growth of HMC-1.1, HMC-1.2 and HMC-1.3 (1H9 and 2E2) in standard growing conditions after 72 h (bar graph; n ≥ 7 experiments; Mean ± SEM) and growth curves over time in full (middle panel) or serum-free media (right panel) (n ≥ 3 experiments; Mean ± SEM) showing HMC-1.3 as the average of both 1H9 and 2E2 subclones. *p<0.05; **p<0.01; ***p<0.001;****p<0.0001 using unpaired t-tests or when indicated with a bracket on the side of the curves, two-way ANOVA. Total cells were counted using a Celigo image cytometer and viable cells were calculated after subtraction of the number of dead cells determined by using PI staining. **(B)** Percent distribution (left) or total cellular expression (right) of KIT protein as determined by FACS analysis where data for HMC-1.3 is the average of both 1H9 and 2E2 subclones. Intracellular KIT was estimated from the difference between total and cell surface. *p<0.05 using an unpaired t-test. Shown is the Mean ± SD. **(C)** Western blot analysis showing constitutive phosphorylation of proteins in prominent oncogenic KIT signaling pathways in the indicated HMC-1 cell lines grown in normal culture conditions. Representative blots are on the left and quantifications of the average relative band intensities are on the bar graphs on the right (Mean ± SD; n ≥ 3 independent experiments). Relative band intensities in each sample were corrected by the relative intensity of β-actin or total protein expression (as indicated) and expressed as fold change compared to HMC-1.1. *p<0.05; **p<0.01 using unpaired t-tests. **(D)** Western blot analysis showing phosphorylation changes induced by 100 ng/mL SCF for the indicated times. Cells were serum starved for 2 h before the addition of SCF. Representative blots are on the left and quantifications of the average changes in relative band intensities are on the right (Mean ± SD of 2 to 3 independent experiments). Relative band intensities in each sample were normalized to the relative intensity of β-actin and expressed as fold change compared to HMC-1.1. Full scans of blots shown in **(C, D)** are shown in **Supplementary Figures 8**, **9**, respectively.

To assess whether the rapid growth characteristics of cultured HMC-1.2 cells could be partly influenced by the presence of V560G in addition to D816V in KIT, we compared the growth rate of the new subline (HMC-1.3) with the parental subline. The two individual HMC-1.3 clones (1H9 and 2E2) grew similarly in liquid cultures (**Figure 2A** and **Supplementary Figure 2**) and thus, for simplicity, the results from subsequent experiments are often shown as averages of both HMC-1.3 subclones. The growth of HMC-1.3 over the course of six days was indistinguishable from that of the parental HMC-1.2 but was greater than for HMC-1.1 cells (**Figure 2A**, middle panel). Unlike HMC-1.1, both HMC-1.2 and HMC-1.3 cells exhibited some ability to expand in the absence of serum (**Figure 2A** right panel), suggesting that although factors in the serum stimulate the growth of all these cell lines irrespective of their mutational status, the presence of D816V-KIT in these cells is sufficient to confer growth capacities in the absence of added factors.

We next examined the potential effects of the reversal of the V560G-KIT mutation on the expression, cellular distribution, and signaling of KIT. Constitutively active KIT is known to accumulate intracellularly due to rapid internalization of the receptor and/or defective transit to the plasma membrane (4, 28–31). FACS analyses indicated that all HMC-1 sublines had similarly increased frequencies of intracellular KIT (solid bar, 50-60%) compared to LAD2 cells, a mast cell line expressing normal KIT (**Figure 2B**). Thus, the KIT variants in HMC-1 cells did not significantly alter the cellular localization of KIT, but the cells with D816V-KIT tended to have a lower proportion of cell surface KIT than HMC-1.1 cells. Total KIT expression measured by FACS analysis was modestly reduced in HMC-1.2 compared to HMC-1.1 or HMC-1.3 cells (**Figure 2B**, right panel). However, this reduced expression was not always detected by Western blot and on average there were no statistically significant differences (**Figure 2C**).

As expected, KIT was constitutively phosphorylated in all HMC-1 sublines but more so in those with D816V-KIT (HMC-1.2 and HMC-1.3 cells) (**Figure 2C**). Similarly, constitutive phosphorylation of AKT, ERK1/2 and STAT5, which reflect, respectively, the activity of the prominent PI3K, MAPK and STAT5 pathways in oncogenic KIT signaling (4, 30, 32), was elevated in both cell lines with D816V compared to HMC-1.1 cells. These results agree with other studies that D816V-KIT is a more potent driver of KIT autophosphorylation and oncogenic signaling in the absence of ligand than cells with only V560G. As reported (33), total STAT5 expression was increased in HMC-1.2 compared to HMC-1.1 cells, but also when compared to HMC-1.3 (**Figure 2C** bottom panels and **Figure 2D**). The expression of STAT5 was thus the only noted difference in signaling proteins between HMC-1.2 and HMC-1.3 cells. However, the ratio of phospho-STAT5 to total STAT5 was similar in both cell lines with D816V (alone or in combination with V560G) (**Figure 2C**).

Cells with gain-of function in KIT are known to appear refractory to SCF (31). The fold change in KIT phosphorylation induced by SCF was more pronounced in HMC-1.1 than in D816V-containing cell lines, which show greater constitutive phosphorylation (**Figures 2C, D**). Nevertheless, at the times examined, the maximal phosphorylation levels induced by SCF in KIT, AKT, ERK, and STAT5 were comparable in all sublines (**Figure 2D**). Note that the quantifications were determined through actin normalization, and thus the apparent higher phosphorylation levels of STAT5 after SCF stimulation in HMC-1.2 are likely due to the elevated STAT5 protein content.

These results demonstrate that HMC-1 mast cells containing D816V KIT alone or in combination with V560G-KIT exhibit similar characteristics in terms of growth, receptor localization, KIT phosphorylation and downstream signal activation in the absence or presence of SCF under the experimental conditions of this study.

### HMC-1.3 cells have distinct transcriptional patterns, altered cell surface markers, and predicted molecular and cellular functions compared to HMC-1.2

**Figure 3:**
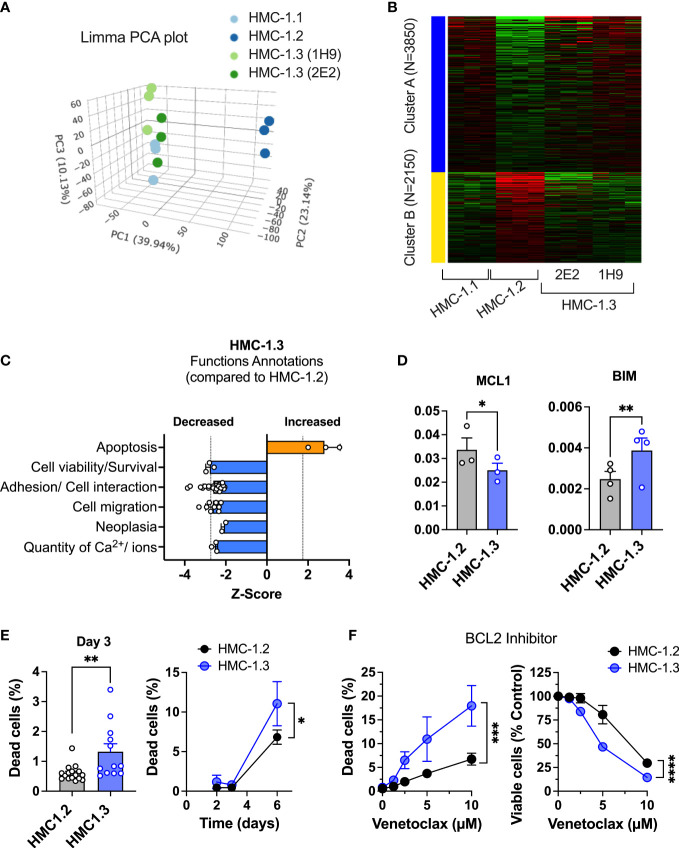
Repair of the G560V-KIT mutation in HMC-1.2 results in distinct transcriptional patterns and reduced pathways that promote survival. **(A)** Principal component analysis plot (Limma) of RNAseq analysis from HMC-1.1, HMC-1.2 and HMC-1.3 subclones illustrating total transcriptional differences between the sublines. **(B)** Heatmap of 6000 differentially expressed genes between the indicated HMC-1 sublines. **(C)** Z-Scores of Functions Annotations predicted to be significantly altered in HMC-1.3 compared to HMC-1.2 using QIAGEN IPA. Each bar represents the average z-scores (Mean ± SD) of similar Functions Annotations terms. These Functional Annotations were categorized by IPA in four top Molecular and Cellular Functions categories: Cellular Movement, Cell Death and Survival, Cell-to-Cell Signaling and Interaction, and Cell Signaling and Molecular transport. **(D)** Relative mRNA expression of the antiapoptotic gene MCL1 and the pro-apoptotic BIM (BCL2L11) in the HMC-1.2 and HMC-1.3 sublines, as indicated. The mRNA levels relative to GAPDH and ACTB (ΔCt) were determined by qPCR. Data are expressed as Mean ± SEM of 3 or 4 independent experiments. * p<0.05; ** p<0.01 using an unpaired t-test. **(E)** Percentage of dead cells in standard growing conditions for 72 h (n=13 experiments; Mean ± SEM) or at days 2, 3, and 6, as indicated (n=3 independent experiments, Mean ± SD). **(F)** Effect of BCL-2 inhibitor (venetoclax) on growth and cell death (n=3 independent experiments; Mean ± SD). Viable and dead cells were determined using PI staining and a Celigo Image Cytometer. Data on HMC-1.3 are the average of both 1H9 and 2E2 subclones. *p<0.05; **p<0.01; ***p<0.001; **** p<0.0001 unpaired t-tests or when indicated with a bracket on the side of the curves, two-way ANOVA.

We next performed transcriptome profiling of the sublines to gain insight into potential differences in cellular activation or function. Transcriptome-wide sample clustering based on the first three principal components (**Figure 3A**) as well as k-means clustering (*k*=2) based on the top 6,000 most variable genes (**Figure 3B**), revealed that the transcriptome of the HMC-1.3 clones (1H9 and 2E2) was unexpectedly distinct from the parental HMC-1.2, and relatively more similar to the HMC-1.1 cells. These observations suggest that despite the similarities observed in the major signaling pathways studied (**Figure 2**), the combined mutations in the JM region (V560G) and KD (D816V) of KIT, have a broader impact on the transcriptome than any of the individual KIT mutations, regardless of the site. Some of the gene expression changes between HMC-1.2 and HMC-1.3 cells included up- or down-regulation in certain cell surface markers aberrantly expressed in malignant mast cells such as CD25, CD44, CD87, and CD2; interleukin receptors including those for IL-7, IL-33, IL-18, and IL-9; and other receptors and adhesion molecules such as CXCR4, CD164, ICAM4, OX40 (CD134), FAS, and SIGLEC8 and 14 (**Table 1**), many of them known to impact mast cell function.

**Table 1:** Expression changes in cell surface markers, receptors, and molecular targets in HMC-1.3 compared to HMC-1.2 cells.

Gene name	FPKM (average of n=3)				Fold Change	*p* value	Fold Change	*p* value
	HMC-1.1	HMC-1.2	HMC-1.3 (2E2)	HMC-1.3 (1H9)	2E2 vs HMC-1.2		1H9 vs HMC-1.2	
CD13 (ANPEP)	121.3	119.0	126.3	129.9	1.02	4.4E-01	-1.08	1.2E-03
CD25 (IL2RA)	37.0	66.2	40.9	34.4	-1.73	3.8E-75	-2.15	3.2E-133
CD33 (SIGLEC3)	48.6	54.5	56.0	52.4	-1.01	8.0E-01	-1.08	6.4E-02
CD44	183.8	340.9	226.8	228.0	-1.53	3.0E-108	-1.70	1.2E-130
CD52	34.0	29.2	50.1	34.6	1.59	4.9E-08	1.12	2.0E-01
CD87 (PLAUR)	27.4	44.3	27.0	28.5	-1.72	2.4E-30	-1.66	6.2E-25
CD117 (KIT)	414.2	355.6	404.56	450.4	1.11	2.7E-09	1.21	8.0E-17
CD32 (FCGR2B)	6.8	15.2	3.70	8.1	-4.16	5.4E-75	-1.92	1.1E-22
CD32 (FCGR2A)	39.5	45.2	36.7	47.8	-1.27	3.7E-11	1.08	3.3E-02
CD59	65.9	50.3	73.5	61.6	1.45	9.6E-34	1.27	3.1E-14
CD63	578.2	552.2	624.7	623.6	1.10	5.1E-04	1.08	1.8E-03
CD66a (CEACAM1)	2.0	3.4	4.1	2.3	1.20	2.7E-02	-1.60	2.0E-07
CD69	2.3	10.1	3.3	2.1	-3.12	7.0E-61	-4.98	7.0E-61
CD71 (TFRC)	50.1	74.9	66.8	41.8	-1.14	3.2E-08	-1.87	6.9E-154
CD95 (FAS)	8.9	5.6	10.9	9.4	1.96	1.3E-28	1.63	5.8E-14
CD116 (CSF2RA)	10.5	10.4	7.4	15.0	-1.39	4.5E-07	1.40	5.9E-09
CD127 (IL7R)	2.7	5.0	3.4	2. 6	-1.51	1.2E-11	-2.10	2.4E-30
CD129 (IL9R)	21.8	9.2	21.4	23.3	2.68	4.9E-66	2.80	1.9E-72
IL1RL1 (ST2)	132.6	205.5	116.5	138.2	-1.90	2.5E-242	-1.60	5.4E-92
CD164	555.4	239.0	398.5	518.0	1.63	2.8E-156	2.05	6.1E-278
CD184 (CXCR4)	0.4	3.0	0.4	0.3	-7.09	4.0E-28	-9.42	1.3E-30
CD203c (ENPP3)	0.3	10.7	0.7	0.5	-17.90	1.9E-193	-23.61	1.8E-188
CD213a1 (IL13RA1)	9.1	9.2	11.7	9.3	1.22	1.9E-06	-1.06	1.8E-01
CD218a (IL18R1)	13.1	16.1	9.7	9.9	-1.69	1.7E-22	-1.70	3.8E-07
CD2	3.4	0.1	0.9	3.3	6.00	5.2E-07	23.38	1.6E-30
CD9	194.3	170.2	235.7	205.2	1.36	2.3E-36	1.16	8.5E-09
CD15 (FUT4)	3.8	4.8	4.2	3.8	-1.17	9.3E-04	-1.32	8.5E-08
CD19	4.2	4.7	2.5	5.3	-2.04	2.9E-07	1.02	8.7E-01
CD48	152.0	92.1	144.8	172.7	1.53	9.8E-48	1.79	2.2E-67
CD50 (ICAM3)	53.7	62.5	53.9	59.7	-1.17	1.4E-06	-1.09	1.0E-02
CD54 (ICAM1)	14.0	14.8	15.4	16.8	1.04	2.9E-01	1.12	4.6E-03
CD58	16.59	14.6	17.7	16.6	1.18	1.3E-02	1.11	1.4E-01
CD134 (TNFRSF4)	1.9	4.5	2.9	2.2	-1.65	1.4E-04	-2.26	1.8E-08
ICAM4	41.9	21.1	37.3	42.1	1.72	1.2E-27	1.90	3.5E-36
ICAM5	8.1	9.0	9.5	9.0	1.00	9.7E-01	-1.02	7.7E-01
SIGLEC14	57.2	37.5	76.6	64.0	2.01	2.1E-107	1.64	2.1E-48
SIGLEC6	283.6	224.7	268.0	309.7	1.14	2.8E-11	1.32	1.2E-29
SIGLEC8	48.0	6.0	21.2	45.1	3.30	6.3E-145	6.70	0.0E+00

Genes in this table were selected based on described cell surface proteins expressed in mastocytosis-like cell lines and/or malignant mast cells in patients with mastocytosis (15). Highlighted in grey are genes whose expression is significantly changed by >1.5 fold in the two HMC-1.3 clones compared to the parental HMC-1.2. Fold changes in red indicate upregulation in HMC-1.3, and in blue, downregulation compared to HMC-1.2.

To better understand the potential molecular and functional implications of these differences in the transcriptional profile, we analyzed differentially expressed genes between HMC-1.3 and the parental HMC-1.2 by Ingenuity Pathway Analysis (IPA). IPA (using genes with a fold change >1.5 or< -1.5, and with FDR<0.05) predicted that functional annotations related to “apoptosis” were increased (Z score=2.8) while those related to “cell viability/survival” were significantly reduced (Z scores< -2) in HMC-1.3 compared to HMC-1.2 cells, suggesting the possibility that the presence of the double mutation (D816V plus V560G-KIT) may confer survival advantages compared to the single mutation under certain conditions (**Figure 3C**).

Genes involved in pathways of apoptosis/survival and reported in association with mastocytosis, such as the BCL2 member myeloid cell leukemia 1 (MCL1) and the pro-apoptotic BCL-2 interacting mediator of cell death (BCL2L11, also known as BIM), were among other differentially expressed genes with fold change >1.2 or <-1.2, and with FDR<0.05, in the comparison of HMC-1.3 versus HMC-1.2 cells (**Supplementary Figure 3A**). MCL1 promotes survival of mast cells and other hematopoietic cells (34, 35) and its downregulation counteracts viability of neoplastic mast cells (36). BIM is suppressed by expression of D816V-KIT (37). We confirmed by qPCR that the relative expression levels of the antiapoptotic MCL1 were significantly reduced in HMC-1.3, while those of the pro-apoptotic BIM were significantly increased compared to the parental cell line (**Figure 3D**). Furthermore, IPA predicted the tumor suppressor protein P53 to be activated (with Z scores >3.1) in HMC-1.3 compared to HMC-1.2 as an upstream regulator. P53 is known to regulate the function of MCL-1 and BIM (38) and alter life/death decisions in mast cells (39–41). The data thus suggest a transcriptional environment in HMC-1.3 less supportive of survival than in HMC-1.2.

In general agreement with this profile, HMC-1.3 consistently showed higher percentages of dead cells in normal culture conditions (**Figure 3E**). However, these percentages were very low (<2%) and hence did not significantly affect the total number of viable cells. Percentages of dead cells were slightly but consistently increased in HMC-1.3 to ~10% after 6 days in normal media (**Figure 3E**) or after two days in serum-free media (**Supplementary Figure 3B**), coinciding with a slight but significant reduction in the number of viable cells (**Supplementary Figure 3C** and middle and right panels of **Figure 2A**) at those time points. To demonstrate whether the reduced survival in the HMC-1.3 cell line may be consequential under stress conditions, we treated the cells with an inhibitor of BCL2, a treatment approved for leukemia that has been shown to promote apoptosis in HMC-1 cells and proposed as a potential therapy for advanced mastocytosis (42). The BCL2 inhibitor venetoclax caused a concentration dependent increase in cell death (**Figure 3F**, left) parallel to a drastic reduction in the number of viable cells (**Figure 3F**, right), and these changes were significantly more pronounced in HMC-1.3 than in HMC-1.2, consistent with the higher propensity of these cells to cell death predicted by IPA.

In addition to cell survival/cell death, functional annotations and molecular functions related to cell-to-cell adhesion, migration and neoplasia were predicted by IPA to be reduced (Z scores<-2) in HMC-1.3 compared to the parental cell line (**Figure 3C**). This was accompanied by changes in the gene expression of adhesion molecules including CD44 (**Table 1**) and other molecules known to be chemoattractant molecules and receptors for mast cells such as CXCR4 (**Table 1**) and CCL2 (or MCP-1) (**Supplementary Figures 3D–F**) and with reported functions in mastocytosis (43–48). Some of these are also depicted in **Supplementary Figure 3D** with predicted upstream regulators by IPA and effects on migration/binding of leukocytes, blood, and tumor cells.

Overall, these results indicate that despite the similarities in KIT levels, intracellular distribution, and constitutive signaling of typical oncogenic KIT pathways between cells with D816V alone or with V560G, the additional presence of V560G causes distinct transcriptional changes including cell surface markers and genes associated with protection against cell death and enhancement of neoplastic/metastatic potential.

### HMC-1.3 cells show reduced transformation potential in a xenograft model and reduced colony formation in soft agar

**Figure 4:**
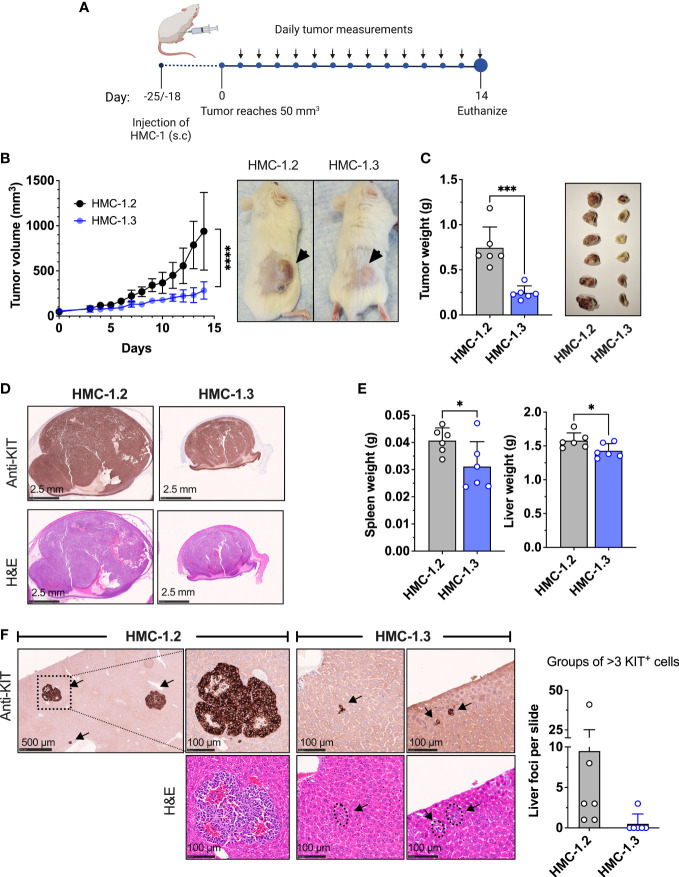
Repair of the V560G-KIT mutation in HMC-1.2 results in diminished tumor growth and metastasis. **(A)** Illustration of the xenograft protocol. Mice (6 per group) were injected with 1 × 10^6^ HMC-1.2 or HMC 1.3 cells (shown is the 1H9 subclone; similar results were obtained using the other subclone 2E2) into the right flank subcutaneously. Once the tumor volume reached 50 mm^3^ (usually 18-25 days after injection), measurements were performed daily using a caliper. On day 14, mice were euthanized for tissue collection. This illustration was created using Biorender (biorender.com) (agreement number BT24ABIPLB). **(B)** Tumor volume measurements on live mice over time. ****p<0.0001 between curves using a two-way ANOVA test. Representative images of the appearance of tumors at endpoint in mice injected with either HMC-1.2 or HMC-1.3 cells. **(C)** Weight of tumors excised on day 14 from mice injected with either HMC-1.2 or HMC-1.3 cells. The images on the right are all fixed tumors collected in this experiment. **(D)** Histology slides of excised tumors from mice injected with HMC-1.2 and HMC-1.3 stained with anti-KIT or H&E, as indicated. **(E)** Average of spleen and liver weights on day 14. **(F)** Representative images of well-formed foci in livers from mice injected with HMC-1.2 cells or groups of >3 KIT positive cells in livers of mice injected with HMC-1.3 cells (indicated by arrows). KIT immunostaining and H&E staining are shown as indicated. The graph represents the numbers of groups of > 3 cells or foci containing KIT-positive cells in the histological slides (each dot represents one mouse). Data represent Mean ± SD. *p<0.05; ***p<0.001 using unpaired t-tests.

Given the IPA prediction of reduced activity in pathways related to neoplasia, migration, and apoptosis/survival in HMC-1.3 compared to HMC-1.2 cells (**Figure 3C**), we used a xenograft model to examine potential differences *in vivo*, under a more physiological environment than the culture conditions. NSG mice were injected with 1x10^6^ HMC-1.2 or -1.3 cells subcutaneously in the flank and allowed to establish an estimated tumor volume of 50 mm^3^ within 18-25 days (**Figure 4A**). Subsequent daily measurements revealed HMC-1.2 cells exhibited accelerated growth, resulting in large, well-formed, and dense tumors, while the injected HMC-1.3 cells were surprisingly slow-growing and resulted in tumors that were significantly smaller in volume and weight and with a soft appearance (**Figures 4B, C**). Histologically, the tumors appeared as a monomorphic mass of KIT positive cells. Other than size, no apparent differences in vascularization/hemorrhage or cellular necrosis were observed between tumors formed by HMC-1.2 and HMC-1.3 cells (**Figure 4D**). We then examined the growth of these cells in colony-forming assays using semi-solid media which provide extracellular scaffolds and thus may be more suitable microenvironment to determine tumorigenicity than the liquid cultures. While both cell lines formed colonies in both soft agar and Matrigel, the colonies formed by HMC-1.3 cells were reduced in number and apparent size compared to those produced by HMC-1.2 cells (**Supplementary Figure 4**), observations consistent with the differences in tumor size *in vivo* (**Figures 4B–D**). These results support a reduced tumorigenicity in HMC-1.3 compared to the parental cells, as also suggested by transcriptome analysis (**Figure 3C**).

To evaluate the metastatic capability into peripheral organs, spleen and liver were also collected during endpoint analysis. These organs collected from HMC-1.2 mice had increased weight compared to those from HMC-1.3 mice, suggesting augmented cellular infiltration (**Figure 4E**). Histological analysis of livers revealed the presence of well-formed tumor foci in mice injected with HMC-1.2 cells (**Figure 4F**), as previously reported (49). In contrast, only one out of six mice injected with HMC-1.3 exhibited three small groups of KIT-positive cells that could represent emerging, but not mature, foci (**Figure 4F**). The spleens of mice injected with HMC-1.2 cells were grossly enlarged compared to those of mice injected with HMC-1.3 cells, mostly evident in the white pulp area (**Supplementary Figure 5A**). However, spleens from neither group developed tumor foci, instead presenting with scattered intense KIT positive staining consistent with the appearance of transformed huMCs, especially in spleens of mice injected with HMC-1.2 cells (**Supplementary Figures 5A, B**).

Overall, these results are consistent with the conclusion that the presence of the two KIT mutations in D816V and V560G confers additional tumorigenicity and metastatic potential.

### HMC-1.3 cells show greater sensitivity to commonly used KIT inhibitors and JAK2 inhibitors than HMC-1.2 cells

Neoplastic mast cell lines are widely employed for the evaluation of new therapeutic targets and testing or validation of new drugs. Since HMC-1.3 cells showed reduced transformation potential (**Figures 4B–F**) and were more susceptible to death-inducing drugs than HMC-1.2 cells (**Figure 3F**), we investigated the impact of the dual or single KIT mutations on the efficacy of drugs that inhibit neoplastic mast cell growth.

**Figure 5:**
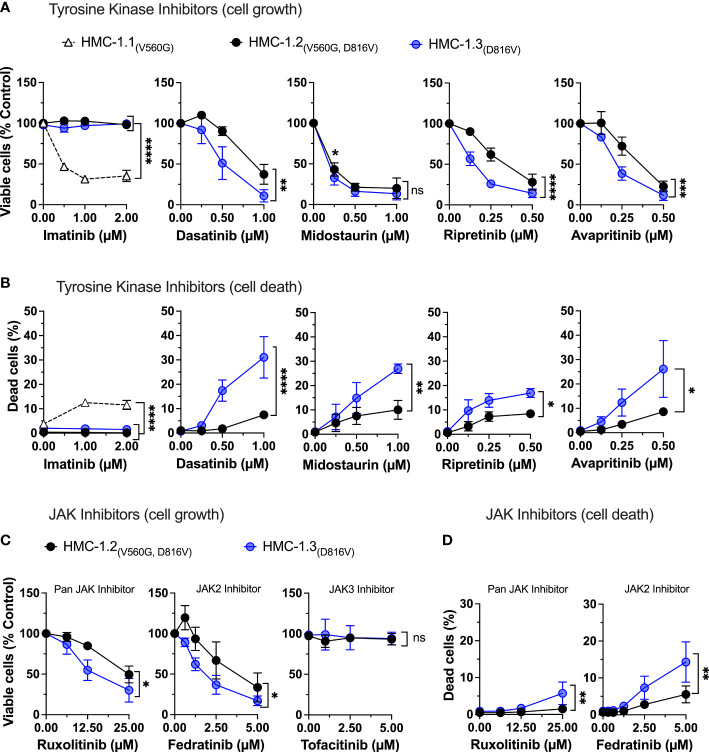
Repair of the G560V-KIT mutation in HMC-1.2 results in higher sensitivity to common tyrosine kinase inhibitors and JAK2 inhibitors. Effects of the indicated tyrosine kinase inhibitors on cell growth **(A)** and cell death **(B)** in HMC-1.2 and HMC-1.3 cells. Graph represents the percentage of viable or dead cells after 72 h in the presence of the indicated concentrations of inhibitors compared to the corresponding cells treated with vehicle (0 µM). Effects of the JAK inhibitors on cell growth **(C)** and cell death **(D)** in HMC-1.2 and HMC-1.3 cells. Graph represents the percentage of viable or dead cells after 72 h in the presence of the indicated concentrations of inhibitors compared to cells treated with vehicle (0 µM). Total cells were counted using a Celigo Image Cytometer and viable cells calculated after subtraction of dead cells which were positive for PI staining. In **(A-D)**, data represent Mean ± SD of 3 independent experiments. ns not significant; *p<0.05; **p<0.01; ***p<0.001; ****p<0.0001 using two-way ANOVA are indicated as brackets between the curves. Note that there were no differences between the curves of inhibition by midostaurin, but at 0.25 µM the percentage inhibition was significantly greater in HMC-1.3 (* p<0.05) using multiple comparison 2-way ANOVA). Data on HMC-1.3 are the average of both 1H9 and 2E2 subclones.

Tyrosine kinase inhibitors are used as a major pharmacological strategy to target KIT activity in KIT-driven diseases but vary in efficacy depending on the type of KIT mutations. Imatinib mesylate (STI571, Gleevec or Glivec) and derivatives effectively inhibit KIT with mutations in the JM domain but are unsuccessful in inhibiting the active conformation resulting from the D816V-KIT mutation. Indeed, the cell lines HMC-1.3 and HMC-1.2 expressing D816V-KIT were resistant to imatinib while HMC-1.1 cells showed significantly reduced cell growth (**Figure 5A**) and increased cell death (**Figure 5B**). In contrast, treatment with other tyrosine kinase inhibitors including dasatinib, midostaurin, the recently developed switch-pocket KIT inhibitor ripretinib and the PDGF receptor inhibitor avapritrinib, effectively reduced the growth of sublines with D816V (HMC-1.2 and HMC-1.3) (**Figure 5A**) as well as cells with V560G-KIT (HMC-1.1) (**Supplementary Figures 6A, B**). This was expected given that these inhibitors effectively block normal and mutated KIT activity, including D816V-KIT. However, HMC-1.3 cells were generally more sensitive than the parental cell line to these inhibitors, particularly dasatinib, avapritinib and ripretinib (**Figure 5A**), which caused a significant increase in cell death in HMC-1.3 (ranging from 15 to 24%) compared to HMC-1.2 (ranging from 5 to 10%) (**Figure 5B**). Thus, although the KIT inhibitors show the expected relative efficacy on both cell lines harboring D816V compared to HMC-1.1, the presence of D816V plus V560G-KIT confers a survival advantage, as cells with a single D816V mutation in KIT are more susceptible than the parental cells.

We also examined the effects of JAK/STAT inhibitors and inhibitors of oncogenic KIT pathways that could hinder neoplastic mast cell growth. In agreement with the known connection of KIT with JAK2 activation, the specific JAK2 inhibitor fedratinib but not a JAK3 inhibitor, and to a lesser extent the pan-JAK inhibitor ruxolitinib, reduced growth in both cell lines. Growth inhibition was more prominent in cells with only D816V-KIT (**Figure 5C**), in association with greater cell death in this subline (**Figure 5D**), similar to cells with only V560G (**Supplementary Figures 6C, D**).

Downstream of JAK, inhibition of STAT3 by C188-9 or inhibition of both STAT3 and STAT5 by a non-selective inhibitor that targets both (SH454), effectively reduced HMC-1.2 and HMC-1.3 growth to a similar extent (**Supplementary Figure 7A**). This was accompanied by a prominent increase in number of dead cells in both cell lines, particularly with the STAT3 inhibitor (30%) (**Supplementary Figure 7B**). Similarly, inhibition of other oncogenic KIT pathways, such as the MAPK/ERK (by U0126) and PI3K/AKT (by LY294002) pathways reduced the number of viable cells similarly in HMC-1.2 and HMC-1.3 (**Supplementary Figure 7C)** in agreement with the similar phosphorylation status of proteins in these pathways (**Figures 2C, D**). The percentage of dead cells trended to be higher in HMC-1.3 in the presence of these inhibitors but generally below 10% and thus had little impact on the overall growth (**Supplementary Figures 7C, D**).

Overall, the engineered HMC-1.3 huMC line containing a single mutation in D816V-KIT is more sensitive than the parental cell line to various KIT and signaling target inhibitors, an effect that appears to be partly the result of increased susceptibility to cell death mechanisms. These findings may have implications for the predictability of the model on drug screening.

## Discussion

A limited number of huMC lines have been established to date, especially those with oncogenic KIT variants that drive human malignancies (i.e, HMC-1 and ROSA^KITD186V^) (12, 15, 50–53). HMC-1 cells containing common KIT variants, isolated from a patient with mast cell leukemia (12), remain one of the most frequent preclinical models to test the efficacy of drugs targeting oncogenic KIT activity and to predict signaling pathways and behavior of neoplastic mast cells (15). In HMC-1.2 cells, KIT genes are composed of a normal and a mutant allele that contains two point mutations, one in the JM region (V560G) and one in the KD (D816V) (13). Heterozygous KIT mutations are representative of the situation in patients with somatic KIT variants but the presence of these two separate KIT point mutations in one KIT allele is rare. Given the complex structural and functional inter-relationship between these regions of KIT (4, 54, 55), it is unclear whether the presence of the two intramolecular mutations may be redundant or result in altered responses. Here we demonstrate, by using CRISPR/Cas9-mediated correction of V560G-KIT in HMC-1.2, that the additional V560G mutation modifies transcriptional programs, molecular components, pharmacological responses, and oncogenic potential in D816V containing cells, features that are important when evaluating potential drug efficacy in D816V-KIT-driven diseases. Thus, the neoplastic huMCs line with a single mono-allelic D816V-KIT described herein (HMC-1.3) provides an improved model of neoplastic huMCs compared to HMC-1.2 and potentially other cell models based on homozygous D816V-KIT, particularly for pharmacological and *in vivo* studies. Furthermore, the results presented herein may help explain the aggressive nature of the HMC-1.2 cell line in contrast to the usually indolent nature of systemic mastocytosis, and resistance to drugs in cells carrying dual alterations in both the JM region and KD isolated from lesions in patients with refractory GIST (18).

The first evidence that the presence of double V560G-KIT and D816V-KIT mutations can have compounding effects came from transcriptome-wide analysis showing that the double KIT mutations have broader effects on the overall transcriptional profile than each mutation independently. Despite the gene expression differences, under our experimental conditions the growth rate in liquid culture, cellular distribution of KIT, and constitutive activation of the KIT oncogenic pathways known to be important for cell growth [such as PI3K, MAPK and STAT5 pathways (1, 4)] were relatively similar between HMC-1.3 and the parental HMC-1.2 cells, but distinct from HMC-1.1 cells. In agreement, inhibition of the PI3K and MAPK pathways or STAT inhibition reduced the growth of HMC-1.2 and HMC-1.3 cells in a similar manner. However, reduced growth and survival between HMC-1.3 and the parental HMC-1.2 cells were unveiled when survival signals such as those derived from KIT activity, BCL2 and JAK2 were blocked by small-molecule inhibitors or when the cells were inoculated *in vivo* in a xenograft model that likely represents a more physiological environment with additional complexities compared to liquid cultures. These may include closer interactions with other cells, extracellular components, and presence of gradients of nutrients, oxygen, metabolites, growth factors, etc., that in total, may help reveal cell behaviors that are masked in the *in vitro* liquid conditions. In support, cell growth assays in semi-solid media, which provide a three-dimensional microenvironment, also reproduced the reduced tumorigenicity of the HMC-1.3 cells observed *in vivo* as they formed fewer and smaller colonies.

The enhanced percentage of dead cells in HMC-1.3 cells in response to the indicated inhibitors also agrees with a transcriptional environment predicted to be less supportive for survival and more conducive to apoptosis than in HMC-1.2, including a predicted increase in P53 activity which is important for the regulation of normal and neoplastic mast cell homeostasis (39–41). Further, these findings are supported by a reduced mRNA expression in HMC-1.3 of the pro-survival MCL-1 protein and increased expression of the pro-apoptotic BIM, both proteins with essential roles in the regulation of mast cell survival/death (34, 36, 37, 56). The differences in gene expression, growth of ectopic tumors or semi-solid media and sensitivity to death by pharmacological agents between HMC-1.2 and HMC-1.3 imply that the combination of the two KIT variants may contribute to other quantitative, qualitative and/or temporal differences in signaling. The higher susceptibility of HMC-1.3 cells to inhibition of JAK2, upstream of the STAT pathways offer JAK2 as one of the potential contributing signaling factors. However, the specific mechanisms and signals for the observed effects may be driven by multiple factors. For instance, potential conformational changes in KIT due to the dual mutations may favor particular KIT dimers among the stochastic combinations of mutated and non-mutated KIT molecules, cause quantitative, qualitative and/or dynamic/temporal changes in certain signaling complexes, or aberrant localization of signals in specific subcellular compartments. Clarification of these aspects would require further investigation.

Notably, our results closely resemble those in another report where point mutations were introduced in the JM Y568 and Y570 of KIT, tyrosines representing docking sites for multiple signaling proteins (54). These mutations, like those described herein, caused an unexpected increase in the transforming capacity of D816V-KIT mutants *in vivo* and modified transcriptional programs *in vitro* but were not accompanied by changes in the PI3K or MAPK signaling pathways or growth in culture (54). Although the KIT constructs in the study using Y568F/Y570F/D816V mutants are distinct to KIT genes with V560G/D816V double mutations, the possibility exists that structural changes imposed by the presence of V560G impedes tyrosine phosphorylation in the JM tyrosine residues or causes equivalent structural alterations, resulting in similar scenarios. Regardless, one conclusion that can be drawn from our results and those from Chaix et al. (54) is that such mutations in the JM region prevent a negative regulatory mechanism on D816V-KIT activity, enhancing its oncogenic potential. Previous reports using *in silico* modeling of KIT showed that a single mutation in the KD such as D816V already causes long range changes in the JM domain that weakens its binding to the KD and prevents its inhibitory role on KIT activity (4, 55), which is consistent with the constitutive KIT signaling observed in cells with D816V (5, 13, 14, 30, 31, 53). However, our study and the other report (54) suggest that the structural changes imposed by the D816V-KIT mutation do not prevent the full inhibitory potential of the JM region which is unveiled when additional KIT mutations such as V560G or Y568F/Y570F are present. This added partial inhibition by the JM domain V560G mutation may also explain in part why single mutations in D816V are considered of low oncogenic potential if not accompanied by other genetic mutations (57–59).

HMC-1.3 cells, as HMC-1.2, injected subcutaneously grew into tumors in a xenotransplantation mouse model, which is a useful feature of both sublines when exploring the efficacy of small molecule inhibitors *in vivo*. However, HMC-1.3 cells not only produced smaller tumors than those produced by HMC-1.2, they also were less metastatic as assessed by the absence of well-formed neoplastic foci in livers of mice injected with HMC-1.3 cells and reduced size of livers and spleens in these mice, indicating globally reduced cell infiltrates. In agreemint with this observation, among the multiple differentially expressed genes between the two cell lines, those related to pathways of cell adhesion, migration, and neoplasia were predicted to be downregulated in HMC-1.3. Thus, it is possible that, in addition to potential differences in the survival or growth of these cells *in vivo*, some of these gene modifications that affect cell migration and their implantation in other niches may contribute to the phenotypical differences in this model. For instance, the mRNA levels of the receptors CD44 and CXCR4, significantly downregulated in HMC-1.3 compared to HMC-1.2 (**Table 1**), are important for homing of stem cells, huMCs and/or huMC progenitors (46–48, 60) and have been associated with hematological malignancies, including systemic mastocytosis (47, 61, 62). Furthermore, similar to the situation in HMC-1.3, downregulation of CD44 expression in neoplastic huMCs reduced mortality in a xenograft model and metastatic cell growth in lungs (47). Another attractive candidate is the chemokine and proangiogenic factor CCL2 (MCP-1), whose mRNA expression was also downregulated in HMC-1.3 compared to HMC-1.2 cells (**Supplementary Figures 3D–F**). CCL2, produced by mast cells in addition to many other cell types and a chemoattractant for mast cells and mast cell progenitors (43, 45, 63, 64) was reported to be elevated in the serum of patients with aggressive mastocytosis, and the levels correlated with poor prognosis (44). Knockdown of CCL2 in neoplastic MCs resulted in reduced tumor growth *in vivo* compared with CCL2-expressing cells and reduced microvessel density in the tumors (44). Thus, although a cause-effect for the reduced expression of these and other genes and the observed phenotype is not established in our study, the reduced tumor growth and metastatic potential of HMC-1.3 align with reports of these proteins as markers of disease severity in mastocytosis.

It is important to note that despite the differences between HMC-1.2 and HMC-1.3, both cell lines containing D816V differ from huMCs cells containing V560G (HMC-1.1 cells) in the general pharmacological profile to inhibitors, growth and constitutive signaling. Thus, both HMC-1.2 and HMC-1.3 were resistant to imatinib, confirming the open conformation of KIT in these sublines, while HMC-1.1 cell growth was compromised by this inhibitor as expected (1, 65, 66). Other tyrosine kinase inhibitors known to target the open conformation of D816V (18, 67–69) were effective in inhibiting the growth of both HMC-1.2 and HMC-1.3 cells albeit at different degrees as they generally caused more cell death in HMC-1.3. In addition, both cell lines with D816V grew faster in culture than HMC-1.1 cells even in the absence of serum where HMC-1.1 cells did not strive, confirming the growth factor-independency of cell lines expressing D816V. Nevertheless, the data also underline that despite the demonstrated usefulness of HMC-1.2 cells as a model of huMCs with D816V-KIT for testing drug efficacy, the combination of the two oncogenic KIT variants has complex effects on cell behavior and survival, thus reducing the predictability of the model. Our findings also support the notion, as other studies have suggested (16, 54, 70), that other double-hit KIT variants, when present in some patient populations with KIT-driven diseases, may not be functionally redundant even if they are gain-of-function and may have unforeseen consequences in pharmacological efficacy studies and molecular cell outcomes.

In summary, in this study we have shown that an intramolecular V560G-KIT mutation, in addition to D816V-KIT, promotes transcriptional programs that lead to increased survival in response to pharmacological inhibitors of KIT and other inhibitors of potential cytoreductive interest and produce smaller tumors with less metastatic capacity, suggesting that in cells with a single D816V-KIT mutation, the JM domain still bears a negative modulatory function. Thus, HMC-1.3 cells modified by CRISPR/Cas9 from the parental HMC-1.2 cell line may represent, in conjunction with the ROSA^KIT D816V^ cell line created by lentiviral transduction of D816V-KIT (53), more representative mast cell models for systemic mastocytosis.

## Data availability statement

The datasets presented in this study can be found in online repositories. The names of the repository/repositoriesand accession number(s) can be found below: https://www.ncbi.nlm.nih.gov/geo/query/acc.cgi?acc=GSE216446.

## Ethics statement

The animal study was reviewed and approved by the NIAID Division of Intramural Research (DIR) Animal Care and Use Committee under the guidance of the Office of Animal Care and Use of the National Institutes of Health, protocol # LAD2E.

## Author contributions

GB and GF designed and conducted experiments, analyzed data, and contributed to the writing of the manuscript. AL, YB, and AP performed experiments and analyzed data. JL processed and analyzed RNASeq data sets. AO supervised the study, designed, and performed experiments, prepared figures, and wrote the manuscript. DM supervised the study and allocated resources. All authors contributed to the article and approved the submitted version.
