# Supplementary material for: CRISPR/Cas9-engineering of HMC-1.2 cells renders a human mast cell line with a single D816V-KIT mutation: An improved preclinical model for research on mastocytosis
Source: Front Immunol. 2023 Mar 21;14:1078958. doi: 10.3389/fimmu.2023.1078958 (PMC10071028; doi:10.3389/fimmu.2023.1078958)
Supplement: Supplementary file 1 [file DataSheet_1.pdf]

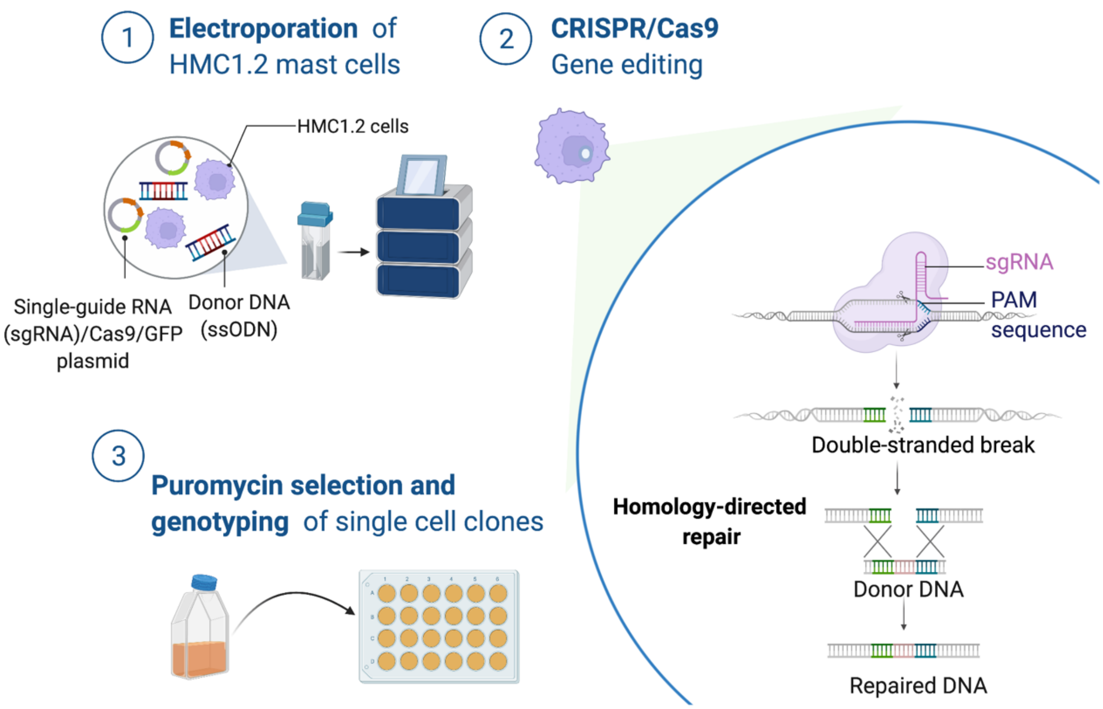

Supplementary Figure 1- Schematic representation of the use of CRISPR/Cas9-targeted editing to repair the V560G mutation in the parental cell line HMC-1.2 harboring both V560G and D816V-KIT. This illustration was created using Biorender (biorender.com) with variations from a template (CRISPR-Cas9 Gene Editing) (agreement number GM246BGEI4).

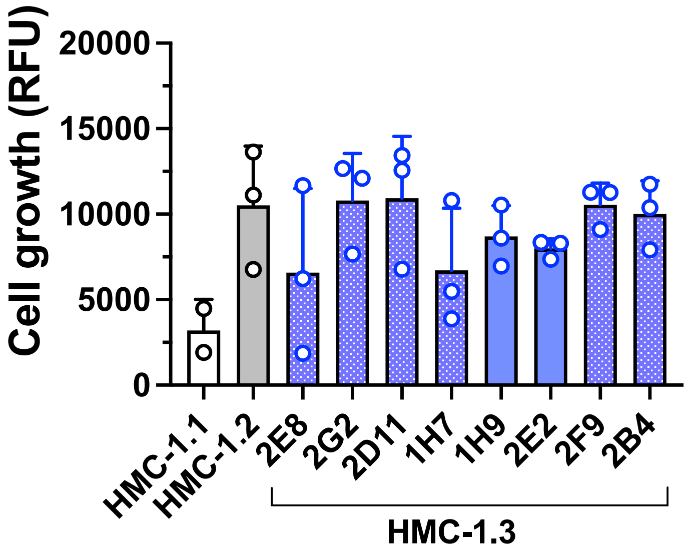

Supplementary Figure 2- Low variations in growth among various HMC-1.3 individual clones. Cell growth of single clones of HMC-1.3 in standard growing conditions after 72 h in culture. Cell growth was determined as fluorescence intensity (relative fluorescence units, RFU) using CyQuant assay. Data is expressed as Mean ± SD of 3 independent experiments, each done in triplicates.

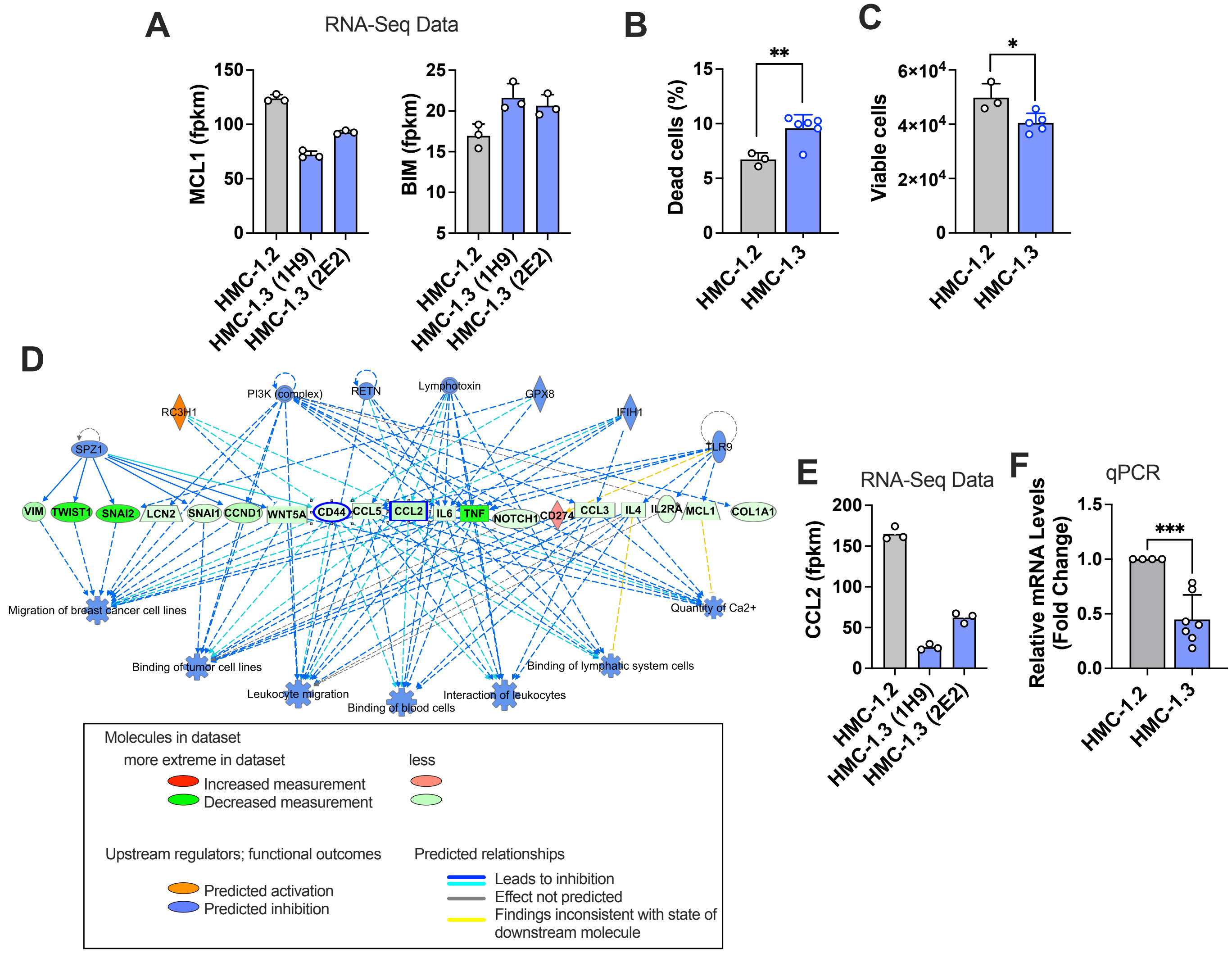

Supplementary Figure 3- Transcriptional changes related to cell survival/death and migration/neoplasia in HMC-1.3 cells compared to HMC-1.2 and differences in cell death and growth in serum free media. (A) Fragments per kilobase of transcript per million mapped reads (fpkm) obtained by RNASeq as described in Methods for MCL1 and BIM (BCL2L11) in HMC-1.2 and the HMC-1.3 clones 1H9 and 2E2. Data represents Mean ± SD. Differences between HMC-1.2 and HMC-1.3 were all with p values<0.0001 and FDR <0.05. (B, C) Cells were plated in serum-free media and counted after 48 h using a Celigo Image Cytometer. The percentages of dead cells (B) were determined by counting cells positive for propidium iodine staining, and the number of viable cells (C) was calculated by subtracting the number of dead cells from the total numbers of cells. Data represents Mean ± SD of 3 independent experiments. *p<0.05 and **p<0.01 using unpaired t-tests. (D) Regulator effects of differentially expressed genes between HMC-1.3 (1H9) and HMC-1.2, connecting predicted upstream regulators (top) and dataset molecules (middle) to downstream functions (bottom) related to migration/adhesion of cancer cells, leukocytes and blood cells. The pathway was generated using QIAGEN IPA (QIAGEN Inc., https://digitalinsights.qiagen.com/IPA). Highlighted are CCL2 and CD44 (bold borders) and the connections of its upstream predicted regulator and functions (light blue arrows). (E) As in A,

fpkm for CCL2 obtained by RNA-Seq. Data represents Mean ± SD. Differences between HMC-1.2 and HMC-1.3 were all with p values<0.0001 and FDR <0.05. (F) Relative mRNA expression of the antiapoptotic gene CCL2 in the HMC-1.2 and 1.3 sublines confirming the RNA Seq data. The mRNA levels relative to GAPDH and ACTB were determined by qPCR and expressed as fold change compared to HMC-1.2 in each experiment (ΔΔ Ct). Data are expressed as Mean ± SEM of 4 independent experiments. *** p<0.001 using an unpaired t-test. Data on HMC-1.3 are the average of both 1H9 and 2E2 subclones.

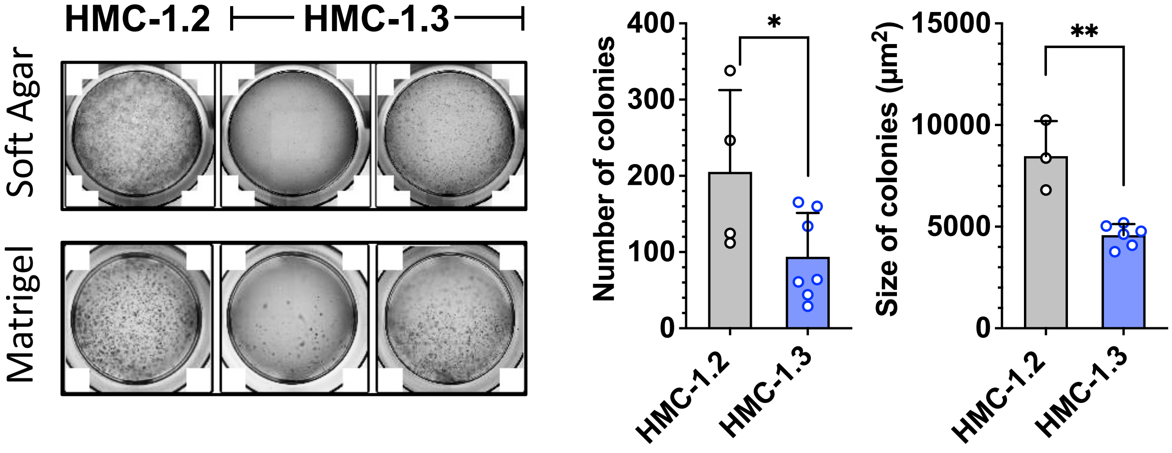

B

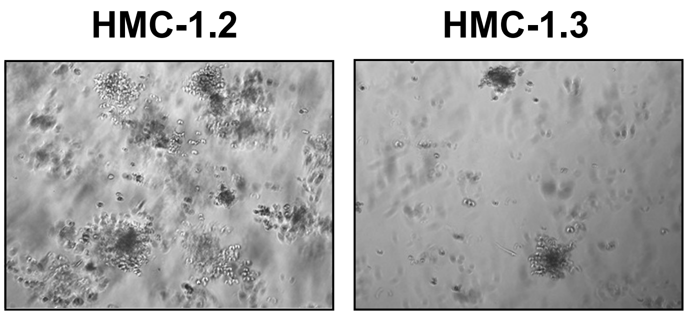

Supplementary Figure 4- Reduced number and size of colonies formed by HMC-1.3 cells in three dimensional cultures. (A) Images of tissue culture wells showing colonies formed by HMC-1.2 and 1.3 cells in soft agar (top) or Matrigel (bottom) obtained using a Celigo imager. Graphs represent the average number of colonies per well in both semi-solid media, and average size after 6 days in culture determined by Celigo ($n \geq 4$). (B) Representative images of colonies in Matrigel (20X) to illustrate the differences in size and appearance between HMC-1.2 and HMC-1.3 grown in three-dimensional cultures. Data represent Mean ± SD; *$p<0.05$; ** $p<0.01$, using unpaired student t-tests.

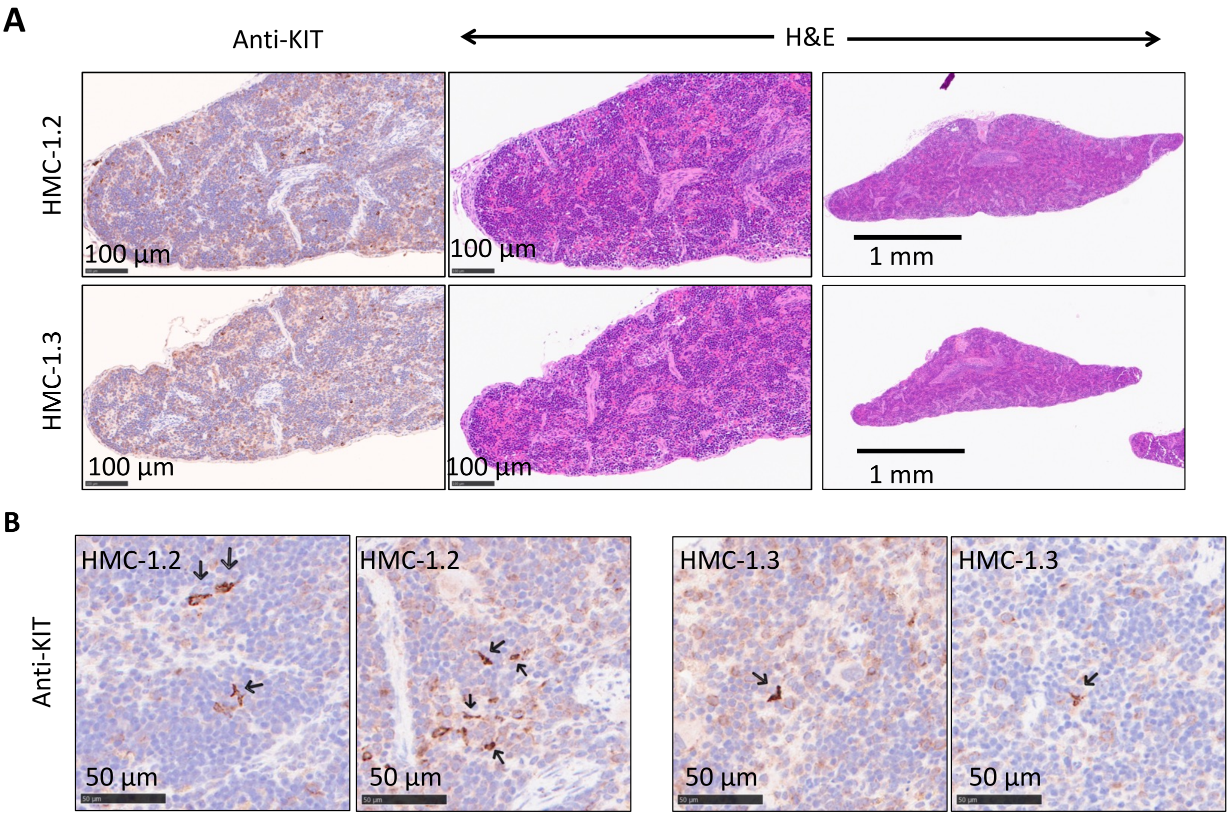

Supplementary Figure 5- Enlarged spleens in mice injected with HMC-1.2 in comparison to those of mice injected with HMC-1.3 cells. (A) Representative images of KIT immunostaining and H&E staining of spleens of mice injected with HMC-1.2 or HMC-1.3 cells, as indicated. Note the overall enlargement of spleens of mice injected with HMC-1.2 cells and larger white pulp (blue in the KIT-staining counterstained with eosin and blue-purple in H&E stained slides). (B) Higher magnification pictures of histopathology samples of spleens stained with anti-KIT. Arrows indicate strong KIT-positive staining consistent with the appearance of the neoplastic huMCs, usually less abundant in spleens of mice injected with HMC-1.3 under the skin.

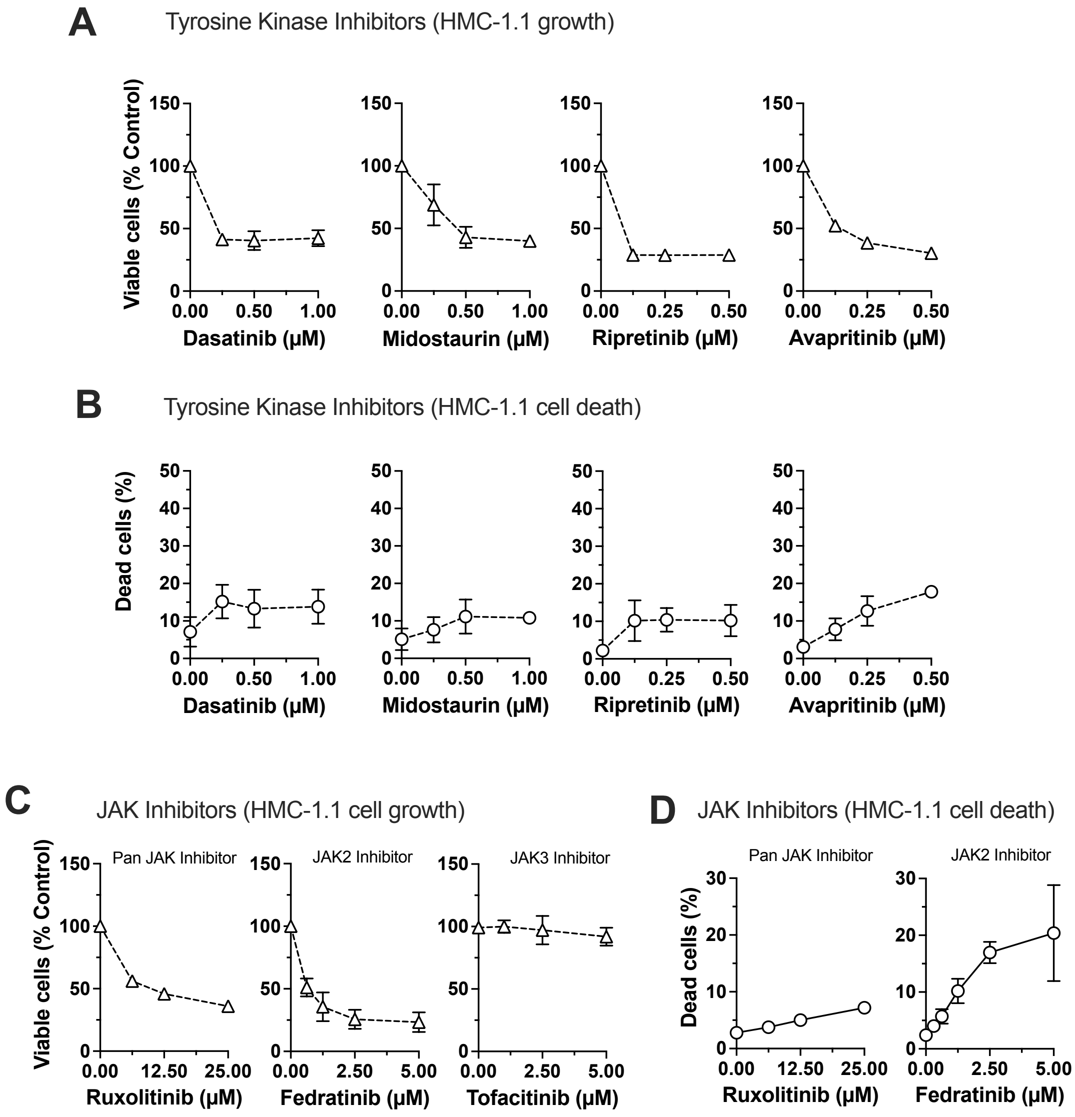

Supplementary Figure 6- Effect of tyrosine kinase and JAK inhibitors on HMC-1.1 cell growth and cell death. The number of viable (A, C) and dead cells (B, D) were determined in HMC-1.1 cultures growing for 72 h in culture media in the presence of the indicated concentrations of inhibitors. Total cells and dead cells stained with propidium iodine were counted using a Celigo Image Cytometer, and the number of viable cells calculated by subtracting the number of dead cells from the total cells. Data are expressed as Mean ± SD of n=3 independent experiments, each done in quadruplicates.

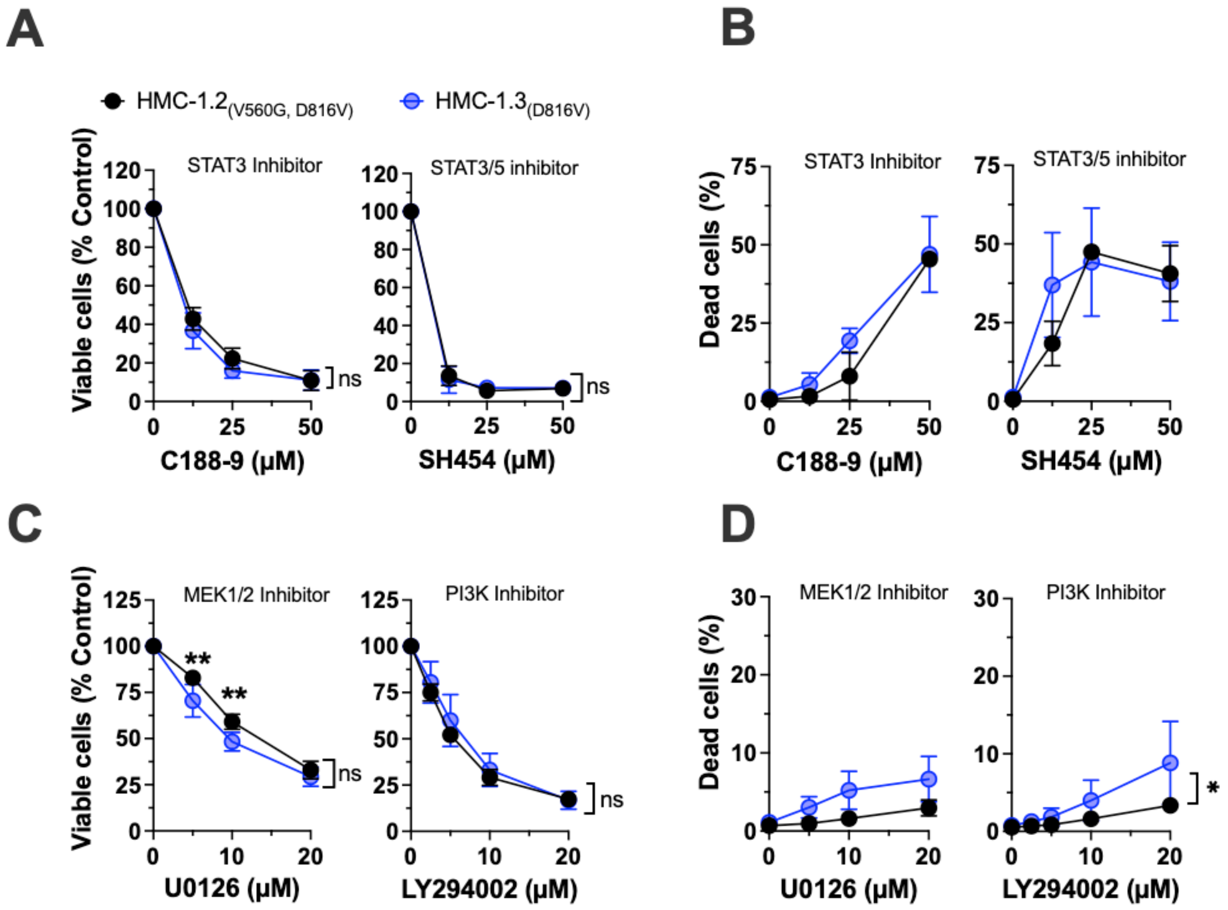

Supplementary Figure 7- Effect of inhibitors of STAT transcription factors, MAPK/ERK and PI3K/AKT oncogenic pathways on HMC-1.2 and HMC-1.3 cell growth and cell death. The number of viable (A, C) and dead cells (B, D) were determined in cultures growing for 72 h in culture media in the presence of the indicated concentrations of inhibitors. Total cells and dead cells (stained with propidium iodine) were counted using a Celigo Image Cytometer and the number of viable cells was calculated by subtracting the number of dead cells from the total numbers of cells. Data are expressed as Mean ± SD of n=3 independent experiments, each done in quadruplicates. n.s., not significant and * $p<0.05$ using two-way ANOVA are indicated as brackets between the curves. When above specific points, asterisks indicate statistical differences between inhibition in HMC-1.2 and HMC-1.3 using unpaired t-tests. ** $p<0.01$ using multiple comparison 2-way ANOVA.

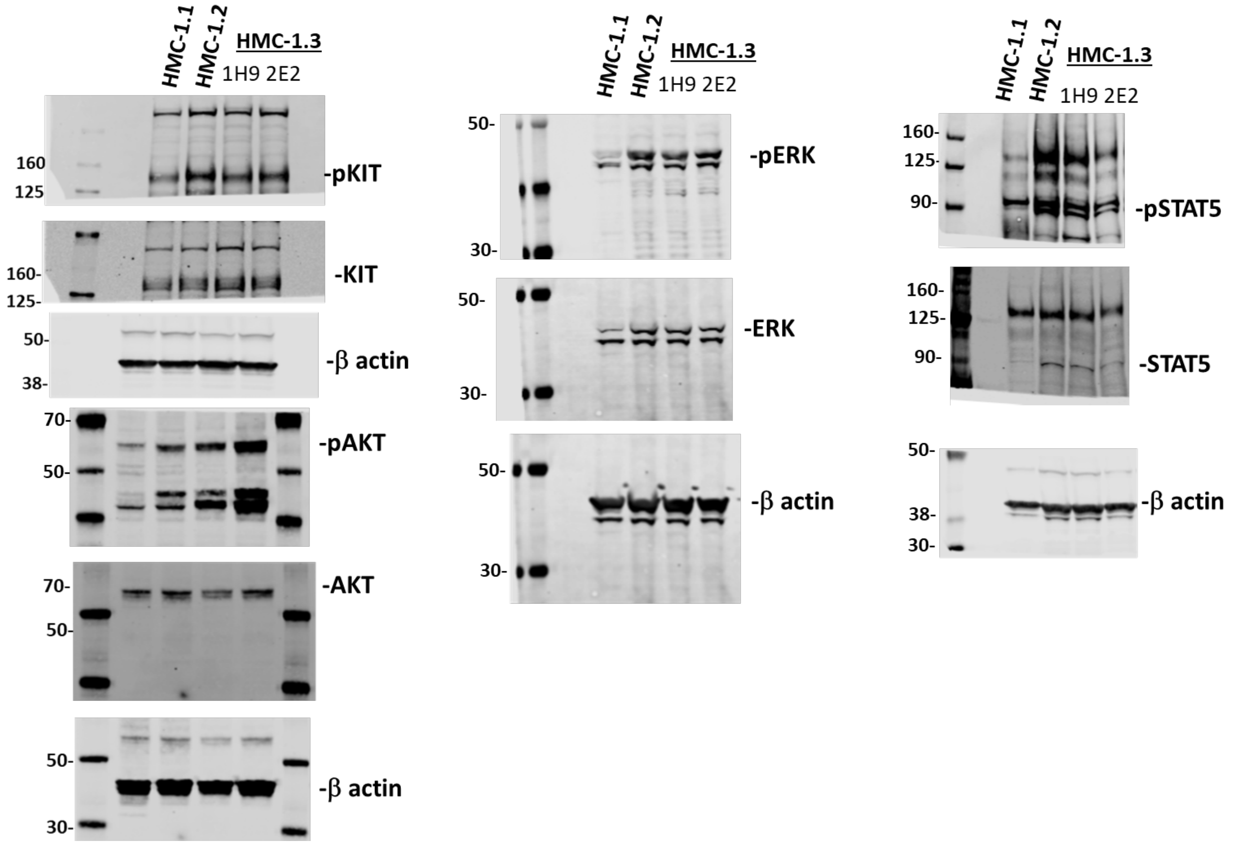

Supplementary Figure 8- Full scans of blots shown in Figure 2C

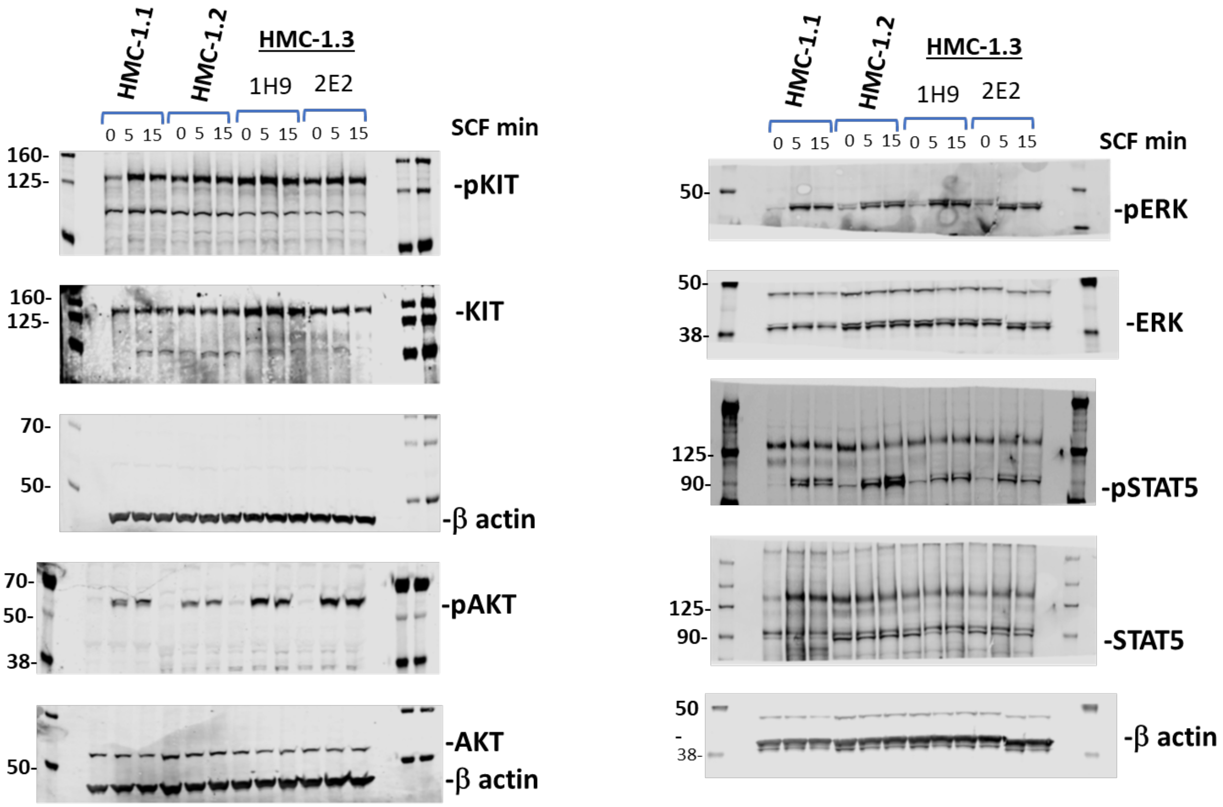

Supplementary Figure 9- Full scans of blots shown in Figure 2D
